# Cholinoceptor Activation Subserving the Effects of Interferon Gamma on the Contractility of Rat Ileum

**DOI:** 10.1155/S0962935194000645

**Published:** 1994

**Authors:** Enri S. Borda, Leonor Sterin-Borda, Martin Rodriguez, Maria M. E. de Bracco

**Affiliations:** 1 CEFYBO-CONICET Serrano 665 1414 Buenos Aires Argentina; 2 IIHEMA Academia Nacional de Medicina Buenos Aires Argentina

## Abstract

Recombinant rat interferon γ stimulated the contractility of isolated rat ileum at doses of 4–12 units/ml. Muscarinic cholinoceptors were involved, as treatment of the tissue with atropine prevented the contractile response of the ileum. Furthermore, interferon γ increased the affinity of carbachol for the cholinoceptors and did not change its maximum effect. Neurogenic pathways were also involved since pretreatment of ileum with hexamethonium, hemicholinium or tetrodotoxin impaired the contractile effect of interferon γ. In contrast to the action of exogenous carbachol, the effects of interferon γ are indirect. They appear to involve a G protein regulating phosphoinositide turnover and cytoskeletal structures since they could not be induced in ileum strips that were pretreated with pertussis toxin, phospholipase C inhibitors (2-nitro-carboxyphenyl, *NN*-diphenyl carbamate and neomycin), cytochalasine B or colchicine.


Mediators of Inflammation 3, 453–458 (1994)

### Cholinoceptor activation subserving the effects of interferon gamma on the contractility of rat ileum

Enri S. Borda,[1,CA] Leonor Sterin-Borda,[1] Martin Rodriguez[1] and Maria M. E. de Bracco[2]

[1]CEFYBO-CONICET, Serrano 665, 1414 Buenos Aires, Argentina; [2]IIHEMA, Academia Nacional de Medicina, Buenos Aires, Argentina

[CA] Corresponding Author

**Recombinant rat interferon γ stimulated the contractility of isolated rat ileum at doses of 4–12 units/ml. Muscarinic cholinoceptors were involved, as treatment of the tissue with atropine prevented the contractile response of the ileum. Furthermore, interferon γ increased the affinity of carbachol for the cholinoceptors and did not change its maximum effect. Neurogenic pathways were also involved since pretreatment of ileum with hexamethonium, hemicholinium or tetrodotoxin impaired the contractile effect of interferon γ. In contrast to the action of exogenous carbachol, the effects of interferon γ are indirect. They appear to involve a G protein regulating phosphoinositide turnover and cytoskeletal structures since they could not be induced in ileum strips that were pretreated with pertussis toxin, phospholipase C inhibitors (2-nitro-carboxyphenyl, *NN*-diphenyl carbamate and neomycin), cytochalasine B or colchicine.**

**Key words:** Cytokines, Cytoskeleton, G Proteins, IFNγ, Intestine, Pertussis toxin, Phosphoinositides

### Introduction

Interferon gamma (IFNγ) is a glycoprotein with antiviral activity that is involved in a variety of immunoregulatory processes.[1] In addition to its effects on immune cell function, it can modulate smooth muscle[2] and intestinal epithelial cell growth.[3] IFNγ is known to induce HLA class II antigen expression on endothelial cells,[4] smooth muscle cells[5] and enterocytes,[6] and this could be important for the induction of an autoimmune or inflammatory response.[7] The intestine normally contains abundant IFNγ-producing T-lymphocytes, and IFNγ release may occur locally and increase after antigen challenge.[8] Through its action on tight junction permeability it could affect intestinal epithelial cell contacts[9] and/or influence the function of the gut by modifying electrolyte transport.[10]

Recently we have shown that recombinant rat IFNγ interacts with isolated rat atria, mimicking the action of a muscarinic cholinergic agonist. Thus, incubation of rat atria with IFNγ decreased tension and cAMP synthesis and increased cGMP production.[11] In this study we investigated if INFγ could also alter the mechanical behaviour of the intestine.

Our results suggest that IFNγ imitates the action of carbachol, a muscarinic cholinergic agent, inducing the contraction of isolated rat ileum. In contrast to the exogenously added agonist carbachol, the effects of IFNγ on ileum also involve a nerve-mediated pathway and are indirect. They require the integrity of the cytoskeleton and involve a regulatory G protein, indicating that IFNγ reaction with the cholinergic receptor is complex and probably involves common signalling pathways.

### Material and Methods

*Drugs and reagents*: Recombinant rat interferon γ (rIFNγ) was purchased from AMGEN Biologicals (CA, USA). Stock solutions containing $10^7$ units/ml were prepared and stored in aliquots at –70°C until used. Fresh dilutions were prepared immediately before use. Blocking experiments with monoclonal anti-rat IFNγ (M anti-IFNγ, Amgen Biologicals, CA, USA) were performed by preincubating rIFNγ (1 000 U/ml) with an equal volume of M anti-IFNγ (10 000 U/ml) during 30 min at 37°C. According to the providers (Amgen Biologicals) the activity of IFNγ (units/ml) was defined by an antiviral assay whereas one unit of M anti-IFNγ was the amount of antibody sufficient for neutralization of one unit of rat IFNγ antiviral activity. The reaction mixture was used thereafter, adjusting the dilution to achieve the final concentration desired in the organ bath. Carbachol, hexamethonium, hemicholinium, tetrodotoxin, 2-nitro-carboxyphenyl, *NN*-diphenyl carbamate (NCDC), neomycin, cytochalasine B, colchicine, pertussis toxin and atropine were obtained from Sigma (St Louis, MI).

*Isolated ileum preparations*: Ileum strips were obtained from male albino rats of the Wistar strain weighing between 200–250 g. The animals were sacrificed by decapitation and after excision of the abdomen coat, the ileum fragments were carefully dissected. They were placed in Petri dishes filled with a modified Krebs–Ringer-bicarbonate (KRB) solution.[11] The ileum was opened longitudinally and small portions (~1 cm long) were used. The ileum preparations were mounted in an organ bath containing 15 ml of KRB solution gassed with 5% $CO_2$ in oxygen and kept at 37°C and pH 7.4. One end of the

© 1994 Rapid Communications of Oxford Ltd tissue was anchored to a stationary glass-holder and the other was connected to a force transducer (Stathan UC3 Gold Cell) coupled to an ink-writing oscillograph (San Ei 180). A constant resting tension of 750 mg was applied to the tissue preparations by means of a micrometric device. Only ileum strips that presented spontaneous contractions were used and the isometric developed tension (in mg) was recorded. Preparations were allowed to equilibrate for 20 min. The contractile tension recorded at this moment (before delivering IFNγ or the drugs) was considered the initial control. These tension values (expressed in mg) were obtained by measuring the amplitude of all the contractions recorded over a 10 min period and calculating their mean value. These initial magnitudes were compared with the experimental values and the variations induced by IFNγ or drugs were expressed as percentage changes with respect to the initial control. The control values of tension at the end of equilibrium and before the addition of IFNγ or drugs was: 200 ± 18 mg ($n$ = 50). Concentration–response curves were constructed according to van Rossum.[12] Single doses were delivered in volumes of 0.01 to 0.025 ml of an appropriate isotonic solution. The total amount of vehicle added to the bath never exceeded 0.25 ml. The time interval between doses was that needed by each dose to produce a maximum effect. Atropine, hexamethonium, hemicholinium, tetrodotoxin, pertussis toxin, neomycin, cytochalasine B, colchicine or NCDC were added to the organ bath 30 min before delivering IFNγ or carbachol. At the concentrations used, these drugs did not alter the baseline.

### Results

IFNγ induced a concentration-dependent increase in the contractile activity of isolated ileum. An original tracing showing the pattern of stimulation is shown in Fig. 1. The stimulatory effect of IFNγ developed gradually, reaching a plateau at 5–10 min. For this reason the ileum was exposed for a period of 8–10 min to each concentration (Figs 1 and 2A). The stimulatory effect of IFNγ lasted for 30–40 min (data not shown). Preincubation of IFNγ with M-anti-IFNγ prevented the reaction (Fig. 2A). To determine whether cholinergic receptors were involved in the action of IFNγ, ileum strips were incubated for 30 min with $10^{-6}$ M atropine before exposure to IFNγ. As shown in Figs 1 and 2A, the positive effect of IFNγ was antagonized by atropine in a non surmountable manner. On the other hand, Fig. 2B shows that atropine antagonized the action of carbachol shifting the dose–response curve to the right (Fig. 2B). Moreover, IFNγ potentiated the action of carbachol by increasing the affinity of the cholinoceptor for its agonist (carbachol) while the maximum effect ($E_{max}$) remained unchanged (Table 1). To determine if the cholinergic IFNγ effect was nerve mediated, hexamethonium ($10^{-6}$ M), hemicholinium ($2 \times 10^{-5}$ M) and tetrodotoxin ($5 \times 10^{-7}$ M) were used as inhibitors. Figure 3A shows a significant reduction of the stimulatory effect of IFNγ when the ileum was preincubated either with the inhibitor of nicotinic cholinoceptors (hexamethonium) or with the acetyl choline (AcCh) synthesis inhibitor (hemicholinium). Likewise, tetrodotoxin, an inhibitor of propagated action potentials, reduced the response of the ileum segment to IFNγ. In contrast, these agents did not modify the response to exogenous carbachol (Fig. 3B). To ascertain whether G regulatory proteins participated in the muscarinic cholinergic action of IFNγ, the ileum strips were treated with pertussis toxin.[13] The results shown in Fig. 4A demonstrate that the stimulatory effect of IFNγ was abrogated by pertussis toxin. In contrast the mechanical response of pertussis toxin-treated tissue to carbachol remained unchanged (Fig. 4B). At least one pathway of cholinoceptor-triggered phosphoinositide is regulated by pertussis toxin-sensitive G proteins that control activation of phospholipase C. To determine if phospholipase C was involved in the cholinergic action of IFNγ we performed experiments in which the phospholipase C activation was inhibited by NCDC[14] or neomycin.[15] The results of Fig. 4A demonstrate that incubation of ileum with NCDC at a concentration of $10^{-6}$ M, which is known to inhibit phospholipase C activity,[14] prevented the effect of IFNγ. On the other hand, NCDC did not alter the inotropic effect of carbachol (Fig. 4B). The same results were obtained preincubating ileum with $10^{-6}$ M neomycin (data not shown). In order to study if cytoskeletal structures were involved in the cholinomimetic action of IFNγ, ileum strips were incubated with the microtubule disrupting agent colchicine, or with cytochalasine B to prevent microfilament polymerization. As shown in Fig. 5A, both colchicine ($10^{-6}$ M) and cytochalasine B ($3 \times 10^{-6}$ M) impaired the stimulatory action of IFNγ. In contrast, neither agent modified the response to carbachol (Fig. 5B) or altered the baseline.

### Discussion

Although most studies on IFNγ activity have focused on the regulation of the immune response, its biological effects are pleiotropic. Many classes of cells bear specific IFNγ receptors and respond to IFNγ binding in different ways.[16–18] We have recently shown that IFNγ triggered metabolic pathways in isolated atria that are considered typical of cholinergic receptor stimulation.[11]

In this study we demonstrate that IFNγ can increase the contractile activity of isolated rat ileum (Figs 1 and 2A). This effect was specific for IFNγ because

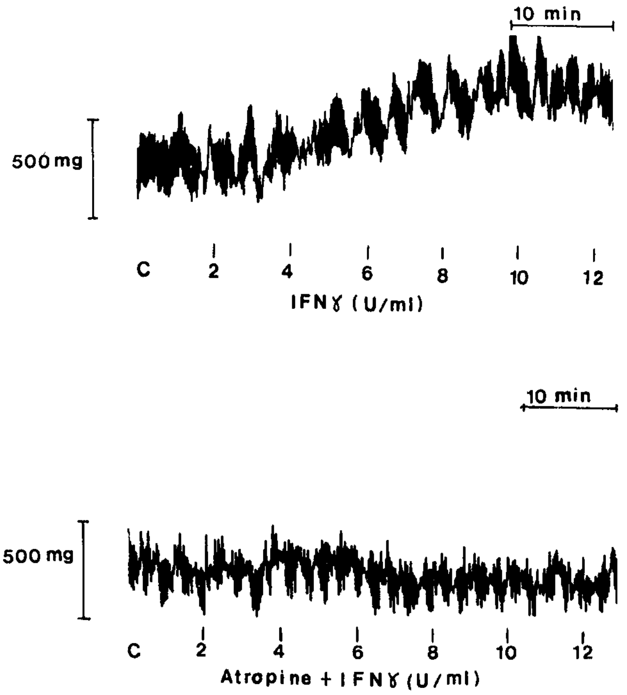

FIG. 1. Original tracings showing the positive inotropic effect recorded from spontaneously contracting rat ileum in the absence (upper panel) or in the presence (lower panel) of $10^{-6}$ M atropine (30 min preincubation) after addition of 2–12 units/ml IFNγ; C: basal values before addition of IFNγ.

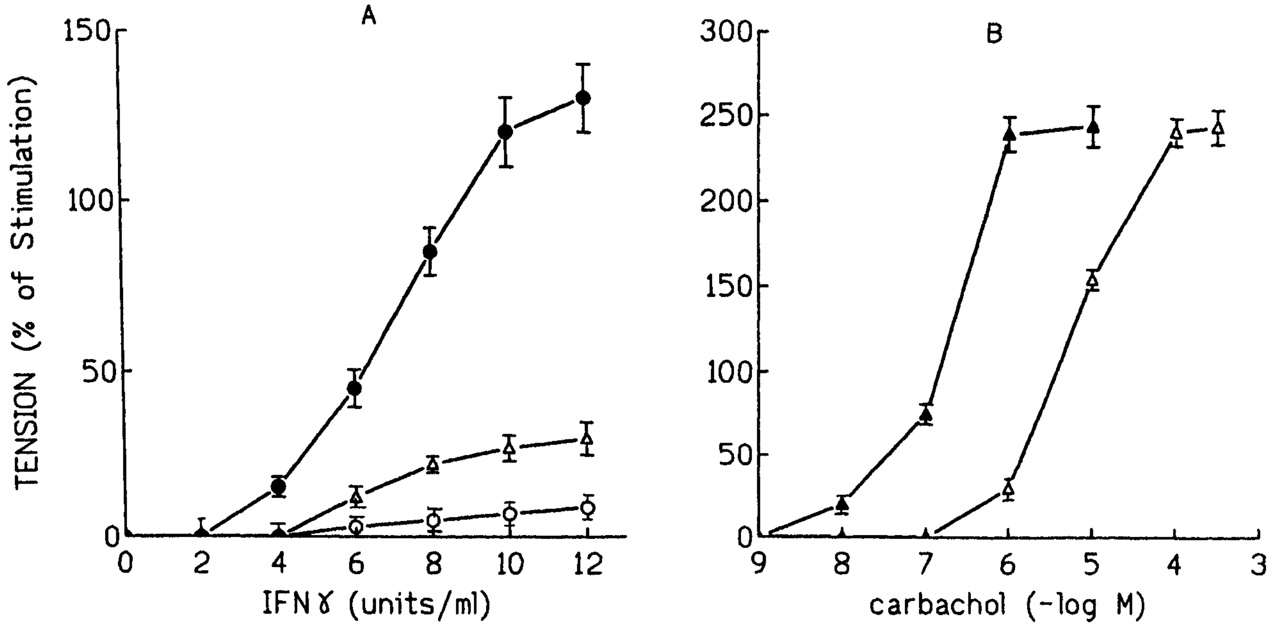

FIG. 2. Effects of IFNγ and carbachol on isolated ileum. Dose–response curves of: (A) IFNγ (units/ml) (●--●) or (B) carbachol (–log M) (▲--▲) were constructed on spontaneously contracting rat ileum or ileum that was preincubated with $10^{-6}$ M atropine (△--△) for 30 min before addition of the reagents. The action of IFNγ neutralized with M anti-IFNγ (O--O) was also assayed. Basal values of tension: 200 ± 18. The results (mean ± SEM) of 12–15 experiments are shown.

**Table 1.** Potentiation of the contractile effect of carbachol by IFNγ

| Additions | $E_{max}$ (mg) | Kd ($10^{-8}$ M) | *n* |
|---|---|---|---|
| Carbachol | 680 ± 40 | 15.0 ± 1.2 | 8 |
| Carbachol + IFNγ | 782 ± 62 | 2.0 ± 0.2* | 5 |

Ileum strips were exposed to different concentrations of carbachol in the absence or in the presence of 2.0 units/ml IFNγ. $E_{max}$: maximum effect of carbachol; Kd: effective concentration of carbachol causing 50% of the maximum response. Basal values: 198 ± 15 mg tension; IFNγ alone: 205 ± 15 mg. Values are mean ± SEM. *n* represents the number of experiments. Statistical differences, carbachol vs. carbachol + IFNγ, * $p < 0.001$, Student's *t*-test.

pretreatment with M anti-IFNγ prevented the action of IFNγ. The contractile response to IFNγ involved muscarinic cholinoceptors, since incubation of the ileum strips with atropine inhibited the reaction in a non-competitive manner. Furthermore, preincubation of the gut with subthreshold doses of IFNγ potentiated the action of carbachol increasing the affinity of the cholinoceptors without alteration of the maximum effect (Table 1). It is noteworthy that the doses of IFNγ required to stimulate ileum tension, and the times of exposure of the tissue to IFNγ, are well below those reported for its action on epithelial cell lines.[9,10] Because IFNγ enhances the fragility of the intestinal epithelial cell barrier, increased peristalsis could facilitate damage of the intestinal function due to the loss of resistance to mechanical stress.[9] The contractile effects of IFNγ appear to be mediated by muscarinic cholinergic mechanisms. However, the effect of IFNγ was impaired by preincubation of the tissue with a nicotinic antagonist (hexamethonium). In contrast to the action of exogenous carbachol at the concentrations used, the contractile effect of IFNγ was shown to involve both myogenic and neurogenic pathways. Nicotinic receptors may be activated directly by IFNγ or indirectly by IFNγ acting on preganglionic nerves or cholinergic interneurones to induce a release of endogenous AcCh, as hemicholinium opposes its action. However, it could be that IFNγ activates cholinergic interneurones which release AcCh to act on nicotinic postsynaptic cholinoceptors; these in turn, could stimulate the release of AcCh to act on smooth muscle receptors. IFNγ induced firing of propagated action potentials, as tetrodotoxin impaired its activity. Nevertheless, as the inhibitory action of tetrodotoxin was only partial, a direct effect of IFNγ on the smooth muscle could also be suggested. Therefore, the interactions of IFNγ with the cholinoceptor pathways of signal transduction appear to be complex. G proteins could be a link between IFNγ and the metabolic pathways triggered by muscarinic cholinergic stimulation. Muscarinic cholinergic receptors (mAcChR) belong to the family of receptors that are coupled to GTP-binding regulatory proteins (G proteins).[19] A single mAcChR can activate more than one type of G protein to regulate several signal transduction pathways.[19] Thus, events that follow muscarinic agonist binding to mAcChR can be the result of pertussis toxin sensitive or insensitive G protein coupled pathways.[20,21] Our results (Fig. 4A) demonstrate that a G regulatory protein is involved in the interaction be-

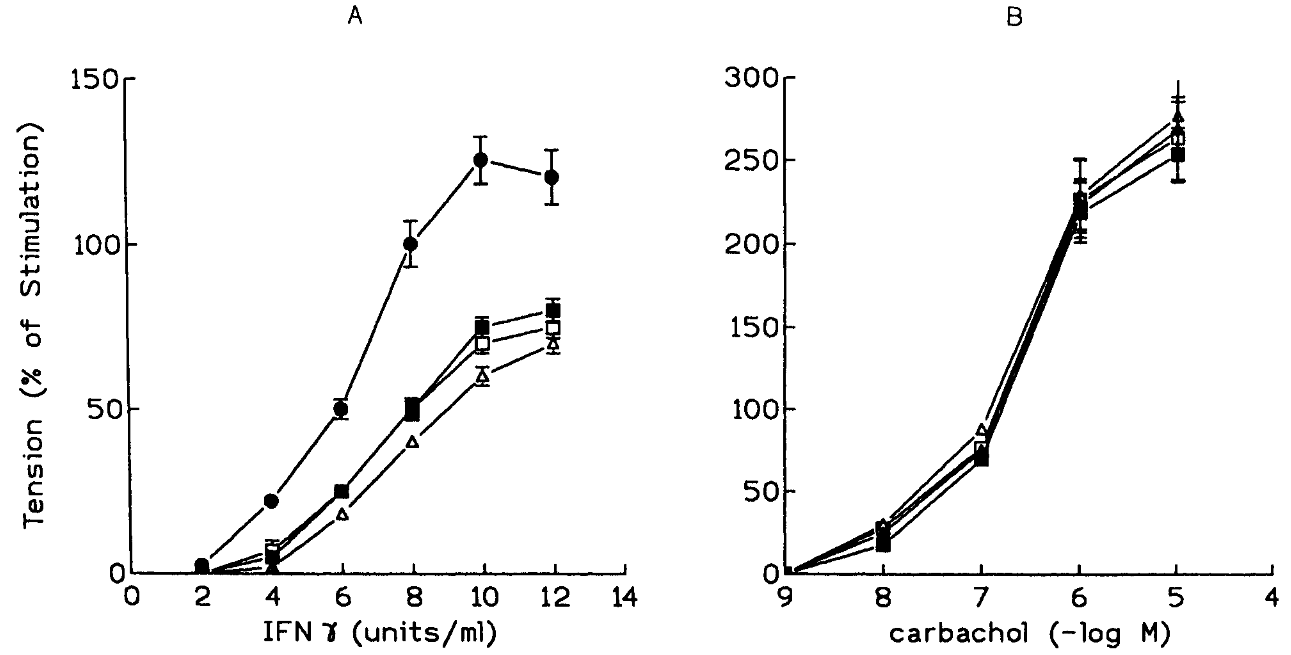

FIG. 3. Participation of the nicotinic cholinergic pathway in the action of IFNγ on rat ileum. Dose–response curves of: (A) IFNγ (●--●) or (B) carbachol (▲--▲) were done on ileum incubated in KRB or preincubated during 30 min with hexamethonium ($10^{-6}$ M) (■--■), hemicholinium ($2 \times 10^{-5}$ M) (□--□) and tetrodotoxin ($5 \times 10^{-7}$ M) (△-△). The results are the mean ± SEM of six experiments.

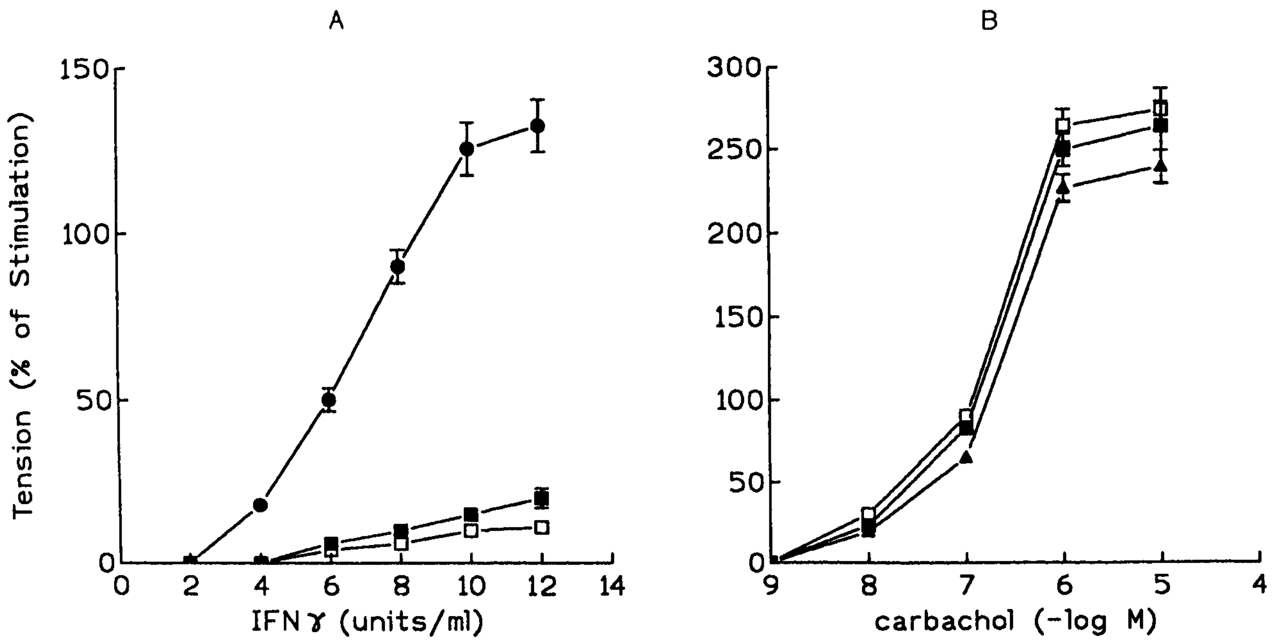

FIG. 4. Participation of G proteins and phosphoinositide hydrolysis in the action of IFNγ on rat ileum. Dose–response curves of: (A) IFNγ (●--●) or (B) carbachol (▲--▲) were done on ileum incubated in KRB or preincubated during 30 min with 0.5 μg/ml pertussis toxin (□--□) or $10^{-6}$ M NCDC (■--■). The results are the mean ± SEM of eight experiments.

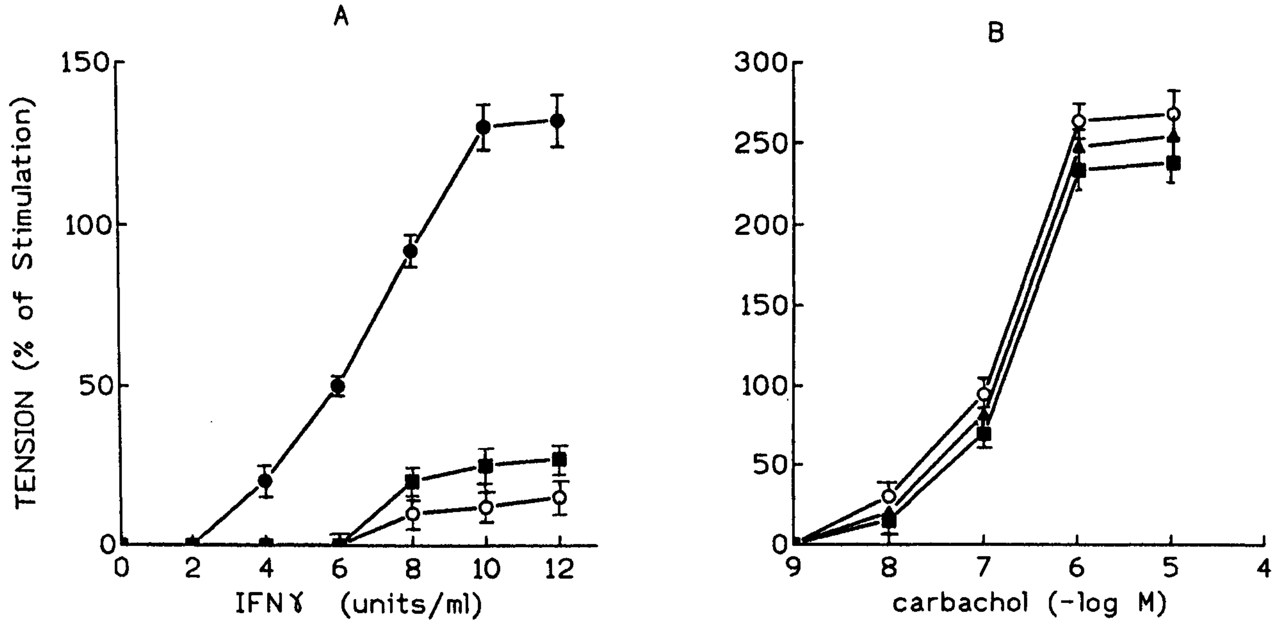

FIG. 5. Participation of the cytoskeleton in the contractile effect of IFNγ on rat ileum. Dose–response curves of: (A) IFNγ (●--●) or (B) carbachol (▲--▲) were done on ileum incubated in KRB or preincubated during 30 min with $10^{-6}$ M colchicine (O--O) or 3 × $10^{-6}$ M cytochalasine B (■--■). The results are the mean ± SEM of seven experiments.

tween IFNγ and the cholinergic system in the intestine, as pertussis toxin treatment of the tissue prevented the reaction. This contrasts with pertussis toxin insensitivity of the carbachol-induced stimulatory effect in the same experimental system (Fig. 4B), suggesting that G protein insensitive pathways predominate in the contractile effect of carbachol. Cholinoceptor activation is associated with phosphoinositide (PI) turnover through phospholipase C activation[13] and smooth muscle contraction can be induced by inositol triphosphate (IP3) resulting from PI hydrolysis.[22] Therefore, we tested if inhibition of phospholipase C could interfere with the cholinomimetic effect of IFNγ on the intestine. Our results demonstrate that PI turnover is required for the development of the IFNγ contractile effect, as NCDC or neomycin treatment of the ileum strips abolished the reaction (Fig. 4A). Again, the stimulatory action of carbachol was insensitive to phospholipase C inhibition (Fig. 4B), indicating that different metabolic pathways resulting in similar mechanical effects may be followed by IFNγ and carbachol. Many reactions mediated by G proteins share common features.[23] Tubulin, a main component of the cytoskeleton, has a GTP binding site and GTPase activity, that are necessary for the polymerization of the microtubules.[23] To determine if the cytoskeleton was involved in the reaction triggered by IFNγ on the ileum, we studied the effect of drugs that interfere with cytoskeletal function (cytochalasine B to prevent microfilament polymerization and colchicine for microtubule disruption) (Fig. 5A and B). While carbachol stimulated contractility under these conditions, IFNγ was ineffective. Thus, the cytoskeleton is probably acting as a link between IFNγ and mAcChR. A fundamental role for the cytoskeleton in the maintenance of the barrier function of epithelial cells has been proposed.[24,25] Direct effects of IFNγ on F-actin distribution have been demonstrated on vascular endothelial cells[18] and in intestinal epithelial cells subtle changes in cytoskeletal rearrangement were observed.[9] However, the amount of IFNγ necessary to obtain these effects was 100 times higher than the dose used to enhance contractility in this study.

In summary, we have shown that IFNγ can trigger the mechanical response of isolated ileum. This is the consequence of a complex and indirect interaction between IFNγ and the cholinergic pathway that involves neurogenic and myogenic pathways, the cytoskeleton, pertussis sensitive G regulatory proteins and PI turnover.

The participation of the gut mucosal immune system in the regulation of intestinal function has been demonstrated.[26] In the normal gut there is a balance in the cytokine network. Under inflammatory conditions or chronic antigenic stimulation at the local level, this balance is disrupted.[27] Lymphokines produced by T-lymphocytes could synergize or antagonize the effects of neurotransmitters or other cytokines[11,28] and thus influence the inflammatory response of the intestine. Thus, the contractile effects of IFNγ described in this study could play a role in chronic inflammatory bowel diseases or in HIV diarrhoea, when the balance of the local immune system is disturbed.[29]

### References

1. Petska S, Langer JA, Zoon KC, Samuel CE. Interferons and their actions. *Annu Rev Biochem* 1987; **56**: 727–777.
2. Warner SJC, Friedman GB, Libby P. Immune interferon inhibits proliferation and induces 2′-5′-oligoadenylate synthetase gene expression in human vascular smooth muscle cells. *J Clin Invest* 1989; **83**: 1174–1182.
3. Cerf-Bensussan N, Quaroni A, Kurnick JT, Bhan AK. Intraepithelial lymphocytes modulate Ia expression by intestinal epithelial cells. *J Immunol* 1984; **132**: 2244–2252.
4. Pober JS, Gimbrone MA, Cotran RS, Reiss C, Burakoff SJ, Fiers W, Ault KA. Ia activation by vascular endothelium is induced by activated T cells and by human γ-interferon. *J Exp Med* 1983; **157**: 1339–1353.
5. Hanson GK, Jonasson L, Holm J, Clowes MM, Clowes AW. γ Interferon regulates vascular smooth muscle proliferation and Ia expression *in vivo* and *in vitro*. *Circulation* 1988; **63**: 712–719.
6. Sollid LM, Gaudernack G, Markussen G, Kvale D, Brandtzaeg P, Thorsby E. Induction of various HLA class II molecules in a human colonic adenocarcinoma cell line. *Scand J Immunol* 1987; **25**: 175–180.
7. Brandtzaeg P, Halstensen TS, Huitfeldt HS, Krajci P, Kvale D, Scott H, Thrane PS. Epithelial expression of HLA secretory component (poly-Ig receptor) and adhesion molecules in the human alimentary tract. *Ann NY Acad Sci* 1992; **664**: 157–179.
8. Targan SK, Kagnoff MF, Brogan MD, Shanahan F. Immunologic mechanisms in intestinal diseases. *Ann Intern Med* 1987; **106**: 853–870.
9. Madara JL, Stafford J. Interferon-γ directly affects barrier function of cultured intestinal epithelial monolayers. *J Clin Invest* 1989; **83**: 724–727.
10. Holmgren J, Fryklund J, Larsson H. Gamma-interferon mediated down-regulation of electrolyte secretion by intestinal epithelial cells: a local mechanism? *Scand J Immunol* 1989; **30**: 499–503.
11. Borda E, Perez-Leiros C, Sterin-Borda L, de Bracco MME. Cholinergic response of isolated rat atria to recombinant rat interferon-γ. *J Neuroimmunol* 1991; **32**: 53–59.
12. van Rossum JM. Cumulative dose–response curves. II. Technique for the making and evaluation of drug parameters. *Archs Int Pharmacodyn Thér* 1963; **143**: 299–305.
13. Brown SL, Brown JH. Muscarinic stimulation of phosphatidyl inositol metabolism in atria. *Mol Pharmacol* 1983; **24**: 351–356.
14. Walenga R, Vanderhoek JJ, Feinstein MR. Serine esterase inhibitors block stimulus-induced mobilization of arachidonic acid and phosphatidylinositide-specific phospholipase C activity in platelets. *J Biol Chem* 1980; **255**: 6024–6027.
15. Serebrinsky G, Isturiz MA. Signal transduction during antibody-dependent cellular cytotoxicity mediated by U937 cells. *Immunol Lett* 1991; **31**: 53–58.
16. Langer J, Petska S. Interferon receptors. *Immunol Today* 1988; **9**: 393–339.
17. Molinas FC, Wietzerbin J, Falcoff E. Human platelets possess receptors for a lymphokine: demonstration of high specific receptors for HuIFN-γ. *J Immunol* 1987; **138**: 802–806.
18. Stolpen A, Guinan EC, Fiers W, Pober JS. Recombinant tumor necrosis factor and immune interferon act singly and in combination to reorganize human vascular endothelial cell monolayers. *Am J Pathol* 1986; **123**: 16–24.
19. Hossey MM. Diversity of structure, signaling and regulation within the family of muscarinic cholinergic receptors. *FASEB J* 1992; **6**: 845–852.
20. Fain JN, Wallace MA, Wojcikiewicz RJH. Evidence of involvement of guanine nucleotide-binding regulatory proteins in the activation of phospholipases by hormones. *FASEB J* 1988; **2**: 2569–2574.
21. Masters SB, Martin MW, Harden TK, Brown JH. Pertussis toxin does not inhibit muscarinic-receptor-mediated phosphoinositide hydrolysis or Ca mobilization. *Biochem J* 1985; **227**: 933–937.
22. Ohta T, Ito S, Noto T, Tachibana R, Nakazaro Y, Ohga A. The inhibitory actions of cAMP on responses to carbachol dependent calcium stores in rat gastric smooth muscle. *J Physiol* 1992; **453**: 367–384.
23. Allende JE. GTP-mediated macromolecular interactions: the common features of different systems. *FASEB J* 1988; **2**: 2356–2367.
24. Meza I, Ibarra G, Sabanero M, Martinez-Palom A, Cereijido M. Occluding junctions and cytoskeletal components in cultured transporting epithelium. *J Cell Biol* 1980; **87**: 746–754.
25. Madara JL. Intestinal absorptive cell tight junctions are linked to cytoskeleton. *Am J Physiol* 1987; **253**: C171–C175.
26. Bienenstock J, Befus AD. Mucosal immunity. *Immunology* 1980; **41**: 249–270.
27. Ciancio MJ, Chang EB. Epithelial secretory responses to inflammation. *Ann NY Acad Sci* 1992; **664**: 210–221.
28. Frohman EM, Vayuvegula B, van den Noort S, Gupta S. Norepinephrine inhibits interferon gamma induced MHC class II expression on cultured brain astrocytes. *J Neuroimmunol* 1988; **17**: 89–101.
29. Greenson JK, Belitsos PC, Yardley JH, Bartlett JG. AIDS enteropathy: occult enteric infections and duodenal mucosal alterations in chronic diarrhoea. *Ann Intern Med* 1991; **114**: 366–372.

ACKNOWLEDGEMENTS: The authors gratefully acknowledge the technical assistance of Ms Elvita Vannucchi. This work was done with funds from CONICET (PID 30066900/88, 3348/92 and 3025000/88).

**Received 25 May 1994; accepted in revised form 8 August 1994**
